# The Role of Nitrosamine (NNK) in Breast Cancer Carcinogenesis

**DOI:** 10.1007/s10911-017-9381-z

**Published:** 2017-06-29

**Authors:** Nomundelger Gankhuyag, Kang-Hoon Lee, Je-Yoel Cho

**Affiliations:** Department of Biochemistry, BK21 PLUS Program for Creative Veterinary Science Research and Research Institute for Veterinary Science, College of Veterinary Medicine, Seoul National University, Seoul, South Korea

**Keywords:** Second hand smoke, Nitrosamines, Breast cancer, Nicotinic acetylcholine receptor

## Abstract

Smoking cigarettes is one of the most concerning issues that leads to tobacco-related cancers and can even result in death. Therefore, these issues should be addressed with a great sense of urgency with low-cost and simple approaches. Over the past several years, the scientific community has attempted to find solutions to overcome this issue. Thus, a large number of excellent studies have been reported in this field, and summarizing these results and providing important roadmaps for future studies is currently of great importance. Finding an outstanding solution to address aforementioned issue would be of great value to the community and to the social. Tobacco contains thousands of chemicals, and sixty-nine compounds have been established as human carcinogens; specifically, 4-(methylnitrosamino)-1-(3-pyridyl)-1-butanone (NNK) is the strongest carcinogen among the tobacco-specific nitrosamines. Tobacco carcinogens are also linked to mammary gland pathogenesis and increased risk of developing many cancers, including breast cancer, the most common cancer in women worldwide. This mini-review summarizes the role of NNK and the mechanisms of its receptor, nicotine acetylcholine receptor (nAChR), signaling in breast cancer based on publications identified using the keywords “secondhand smoke (SHS)”, “Nitrosamines” and “breast cancer”. Furthermore, this review considers the risk of NNK to the public in an effort to reduce exposure to SHS in women and their chances of developing breast cancer.

## Introduction

The effect of smoking tobacco has been studied in patients with lung cancer or smoking-related cancer, including breast cancer, due to heavy smoking [1, 2]. Breast cancer is the most common cancer in women; 1.7 million were diagnosed and 521,000 died worldwide in 2012 [3, 4]. Smoking tobacco has been suspected as one of the risk factors which can be regulated for breast [5, 6].

However, many recently published studies show that the risk of developing breast cancer is not directly associated with active smoking. Since 1986, an expert panel agency that includes the US Surgeon General and the International Agency for Research on Cancer (IARC) repeatedly concluded that the evidence does not support an association between smoking and the risk for breast cancer [7, 8]. Moreover, Hamajima et al. concluded that active smoking and breast cancer did not correlate based on their collated analysis of 53 epidemiological studies that included more than 150,000 cases [7].

On the other hand, secondhand smoke (SHS) (also known as passive smoking, involuntary smoking and environmental tobacco smoke (ETS)) has been identified as a toxic air contaminant since 2006 by Air Resources Board, California Environmental Protection Agency [9]. Non-smokers, who inhaled tobacco gases by being exposed to environmental tobacco smoke, are called passive and secondhand smokers. Throughout life, SHS occurs in the childhood household, in the adulthood household, and at work [10]. The CAREX (carcinogen exposure) database confirmed that in the European Union, 7.5 million workers are exposed to environmental tobacco smoke during ~75% of their workday, which is the second-most common environmental hazard after solar radiation (9.1 million workers) (http://www.ttl.fi/Internet/English/Organization/Collaboration/Carex/default.htm) [11]. Worse than active smoking, the association of SHS with the risk of breast cancer has been contested because the assessment of SHS is more difficult than that of active smoking due to lack of information about a major source of exposure [12, 13]. Notably, recent studies of large number of prospective cohorts provided evidence to suggest that breast cancer risk is elevated among women exposed to the highest levels of SHS [14]. In addition, a study by Johnson and Glantz comparing the strength of evidence from epidemiologic studies showed that SHS affects breast cancer more significantly than lung cancer [15].

Tobacco smoke is well known to contain thousands of chemicals, including more than 60 known carcinogens, which cause a variety of diseases and cancers [16]. Out of 60, at least 20 chemicals have been documented to induce mammary tumors in animal studies using rodent models [17] (Table 1). For the last few decades, 4-(methylnitrosamino)-1-(3-pyridyl)-1-butanone (NNK) has been implicated in the risk of breast cancer development, it remains unclear in breast cancer development, specifically breast cancer induced by SHS [18, 19]. Moreover, NNK can reportedly bind to and activate nicotine acetylcholine receptors (nAchRs) [20, 21], and cancer cells express nAChRs, suggesting that these receptors play a crucial role in cancer development [22].

**Table 1:** Summary of chemicals associated with increased mammary gland tumors in rodent models

Chemicals	Concentration in smoke of nonfilter cigarette	IARC group
benzo(a)pyrene	20–40 ng	2A
toluidine	30-337 ng	2B
4-aminobiphenyl N-heterocylic amines	25–260	1
IQ	0.3	2A
hydrazine	24–43	2B
PhIP	11–23	2B
isoprene	450–1000 I g	2B
benzene	20–70 I g	1
styrene	10 I g	2B
nitromethane	0.5–0.6 I g	2B
nitrobenzen	25 Ig	2B
acrylamide	Present	2B
acrylonitrile	3–15 I g	2A
vinyl chloride	11–15 I g	1
ethylene oxide	7 I g	1
propylene oxide	0-100 ng	2B
dibenz(a,h)anthracene	4 ng	2A
***N-nitrosamines***		
N-nitrosonornicotine	120–3700 ng	2B
N-nitrosodimethylamine	2–1000 ng	2A
N-nitrosodiethylamine	ND-2.8 ng	2A
4-(methylnitrosamino)-1-(3-pyridyl)-1-butanone	80–770 ng	2B

IQ: 3-methylimidazo[4,5-fjquinoline, PhIP: 2-Amino-1-Methyl-6-Phenylimidazo 4 5-b pyridine

Although active and passive smoke likely contain same chemicals but at different amounts, the fact that SHS is significantly more toxic than active smoke is surprising [23, 24]. This review focuses on the role of NNK in SHS-triggered breast cancer and the molecular mechanism underlying NNK-induced carcinogenesis.

## Tobacco Nitrosoamines

**Fig. 1:**
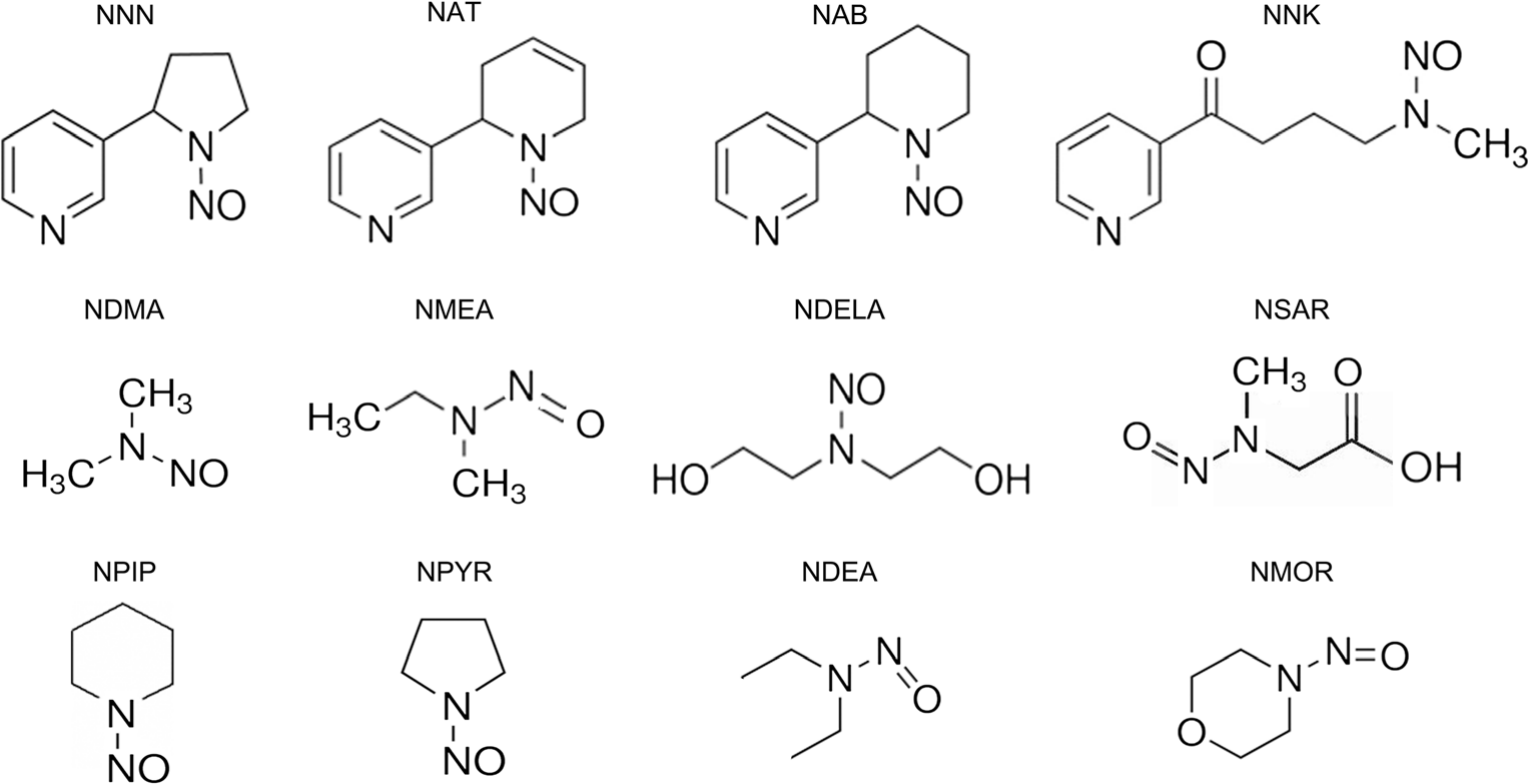
**The structures of the nitrosoamines found in tobacco smoke.** NNN (N-nitrosonornicotine), NAT (N-nitrosoanatabine), NAB (N-nitrosoanabasine), NNK (4-methylnitrosamino-1-3-pyridyl-1-butanone), NDMA (N-nitrosodimethylamine), NMEA (N-nitrosomethylamin), NDELA (N-Nitrosodiethanolamine), NSAR (N-nitrososarcosine), NPIP (N-nitrosopiperidine), NPYR (N-nitrosopyrrolidine), NDEA (N-nitrosodiethylamine), NMOR (N-nitrosomorpholine)

More than 70 tobacco species are known; tobacco is a green and leafy plant that originates in South America and contains nicotine, its main addictive compound, and thousands of other chemicals. Therefore, tobacco is often referred to as a chemical factory. The major chemical components of tobacco are converted to 5300 tobacco smoke compounds, such as nicotine derived N-nitrosamines, polycyclic aromatic hydrocarbons (PAHs), aromatic amines, aldehydes and other inorganic and organic compounds [25]. Roberts classified unburned tobacco and cigarette smoke into a functional group of 25 classes of chemicals, and Hoffmann and coworkers subsequently added N-nitrosamines to this group. Gudzinowicz identified more chemical substances in tobacco leaf in 1980 [26]. The US Food and Drug Administration (FDA) and “Hoffman analytes” lists both have identified “harmful and potentially harmful” constituents in tobacco products, including two classes of compounds that have garnered significant attention: polycyclic aromatic hydrocarbons, mainly benzo[*a*]pyrene, and Nitrosamines (N-nitrosodiethylamine (NDEA), N-nitrosodiethanolamine (NDELA), N-nitrosodimethylamine (NDMA), N-nitrosomethylethylamine (NMEA), N-nitrosomorpholine (NMOR), 4-(methylnitrosamino)- 1-(3-pyridyl)-1-butanone (NNK), N-nitrosonornicotine (NNN), N-nitrosopiperidine (NPIP), N-nitrosopyrrolidine (NPYR), and N-nitrososarcosine (NSAR)), especially NNK [27, 28]. (Fig. 1). Some of these chemical compounds, which are released when tobacco is burned, are carcinogenic and toxic [29]. Their carcinogenicity has been extensively examined both in vivo and in vitro systems [30–32]. Tobacco-specific *N*-nitrosamines, such as NNK, are derived from tobacco curing and smoking and their levels range from 50 to 200 ng per cigarette [33, 34].

## Tobacco-Specific Carcinogen Nitrosamine, NNK

As mentioned above, nicotine is an addictive but non-carcinogenic to healthy laboratory animals [33], and nicotine has consequently not been significantly researched in the context of cancer. However, the nitrosation of nicotine and alkaloids results in a “nicotine-derived nitrosamine ketone”, 4-(methylnitrosamino)-1-(3-pyridyl)-1-butanone (NNK) [35], by replacing the N-H moiety with an N-N = O moiety, a change that was first reported in 1978 [30]. Among tobacco-specific nitrosamines, NNK is widely considered the most potent carcinogen to humans for tobacco-induced cancers and was evaluated by the International Agency for Research on Cancer [10, 36]. NNK has been detected in cigarettes, cigars, snuff tobacco and mainstream and sidestream tobacco smoke. Nitrosamines affect not only tobacco consumer but also non-smokers, who are exposed to environmental tobacco pollution [37]. Moreover, tobacco-specific nitrosamine, 63–74% of which consists of NNK, is formed from nicotine during smoking [38, 39](Table 2). Nicotine-derived nitrosamines can cause cancers or increase the risk of cancer in animals and humans [40–43], and humans may be exposed to nitrosamines by inhaling both mainstream and sidestream smoke [44]. Accordingly, carcinogenic NNK and its metabolite are found more in the urine (0.06 to 1.4 pmol/mg creatinine) and blood (42 ± 22 fmol/mL) of smokers than urine (0.02 to 1.2 pmol/mg creatinine) and blood (<8 fmol/mL) of nonsmoker [45, 46]. Current technical improvements have also directly detected nitrosamine-hemoglobin adducts in the blood of smokers [47]. Notably, raw sidestream smoke includes 20–30-fold more aromatic amines and 30–90% more particulate matter than mainstream smoke [48]. In addition, worldwide use of electronic cigarettes has increased rapidly. It is known that electronic cigarette liquids contain low levels of tobacco-specific nitrosamines but a few studies have tested that vapour from electronic cigarette is not free from NNK. Goniewicz et al. analyzed some carcinogens and toxicants in vapour from electronic cigarettes and found NNN (0.08–0.43 ng/15puffs) and NNK (0.11–2.83 ng/15puffs smoke [49]. Farsalinos et al. also reported NAB at trace levels (1.2 ~ 2.3 ng/g) in vapour from electronic cigarettes [50].

**Table 2:** Tobacco-specific nitrosamines in both smokeless tobacco and cigarette smoke

Smoke type	NNN	NNK	NAB	Reference
Electronic cigarette (ng/15puffs)	7.7 0.00008–0.00043	ND 0.00011–0.00283	1.2–2.3 ng/g	Cressey [117] Grana [118]
Others (chewing, etc.) (ppm)	0.47–64	0.03–14	0.03–6.5	Hecht and Hoffmann [77]
Mainstream smoke (μg/cigarette)	0.33–12.454	0.004–4.2	0.33–4.6	Hecht and Hoffmann [77], Fisher et al. [119], Hoffmann et al. [120]
Sidestream smoke (μg /cigarette)	0.15–16.6	0.39–15.7	0.15–1.5	Hecht and Hoffmann [77] Hoffmann et al. [120]

NNN: N-nitrosonornicotine, NNK: 4-(methylnitrosamino)- 1-(3-pyridyl)-1-butanone, NNB: N-nitrosoanabasine

## SHS and Breast Cancer

In fact, true non-smokers may not exist since all individuals are exposed to SHS at some point during their life, either in the childhood household, in the adulthood household, or at work [10]. In other words, most non-active smokers willingly or unwillingly inhale tobacco smoke from the environment. Moreover, previous meta-analysis studies have reported that breast cancer is associated with cigarette smoking because long periods of exposure to SHS increased breast cancer risk [2, 51, 52]. A.Wesley Horton reported that breast cancer and cigarette consumption are closely related in 20 countries, and the inhalation of tobacco smoke is considered a major environmental risk factor for breast cancer [14]. Furthermore, Chu et al. found that cigarette smoking significantly increases the risk of breast cancer in younger or premenopausal women, and this risk strongly correlates with smoking intensity and duration [53]. Similar studies have been reported in several countries, including Japan, Canada, and the USA [8, 54–56]. The main risk of cigarette smoking-associated breast cancer is presumably related to exposure to the potent carcinogens contained in tobacco smoke, including aromatic hydrocarbons, aromatic amines and nitrosamines, which may cause DNA mutations and DNA adducts [57]. Thus, many meta-analyses evaluated causal associations between environmental tobacco smoke and breast cancer and were reviewed by the California Environmental Protection Agency [58]. These studies demonstrated a causal relationship between exposure to SHS and breast cancer [59].

Reynolds et al. reported that high levels of SHS during adulthood may increase the risk of breast cancer in female lifetime non-smokers exhibiting the highest level of cumulative exposure (HR, 1.18; 95% CI, 1.00–1.40) [12]. Furthermore, breast cancer risk was strongly associated with SHS, and SHS exposure is reportedly riskier than active smoking [60]. Schick and Glant investigated unpublished in vivo research data on SHS obtained by the cigarette industry. Interestingly, animal experiments showed that inhaled sidestream smoke is approximately four times more toxic than mainstream cigarette smoke. Sidestream smoke can also damage the respiratory epithelium after 21 days of exposure, and the length of exposure directly correlates with the degree of damage. Overall, the study showed that whole sidestream smoke is more toxic than all of its major constituents combined [61]. Passive exposure is recognized as a high-risk factor because N-nitrosamines and carcinogens found in tobacco are more concentrated in sidestream than in mainstream smoke [10, 61]. Brunnemann et al. quantitatively analyzed N-nitrosamines in tobacco, fresh cigarette mainstream smoke and sidestream smoke using a thermal energy analyzer. Specifically, the representative nitrosamines in tobacco smoke, such as dimethylnitrosamine, methylethylnitrosamine, diethylnitrosamine, and nitrosopyrrolidine, were found at low levels (0.1 ng–97 ng) in mainstream smoke and higher concentrations (9 ~ 1770 ng) in sidestream smoke [62].

In cohort studies, Luo et al. observed that passive smokers who had never been active smokers had a 32% increased risk of breast cancer [63]. A relationship was also identified between breast cancer and the husband’s smoking habit among Korean and Chinese women. Specifically, the risk directly correlated with the duration of the husband’s smoking habit [64, 65]. In Thailand, sources of passive tobacco exposure include spouses (40.8%), the workplace (30.6%), and public areas (26.3%), and this exposure is an important risk factor for breast cancer among females in urban areas [66]. Moreover, an ecologic analysis indicated that women who live in smoke-free environments had a lower risk of breast cancer [67], and importantly, scientific data show that passive smoke is the most important risk factor for female breast cancer, despite the fact that passive smoke is more diluted than direct smoke [15]. These results suggest that effective preventative methods, such as smoke-free public environments, are needed to prevent breast cancer. Since 1985, many research groups have published relationships between breast cancer and SHS, as summarized in Table 3, which shows the results of 10 prospective cohort studies, 16 case-control studies and 7 meta-analyses.

**Table 3:** Studies of secondhand smoking and breast cancer

Study	Study Population (number of participants)		Follow-up (years)	SHS Exposure	Relative Risk of Breast Cancer in Women Exposed to SHS Compared to Women Not Exposed to SHS, RR (95% CI)
Prospective cohort studies					
Egan et al. [121]	78,206 (3140)		14	home or at work	0.90 (0.67–1.22)
Reynolds et al. [122]	116,544 (2005 cases)		5	home	0.94 (0.82–1.07)
Pirie et al. [123]	224,917 (2518 cases)		4	home	1.02 (0.86–1.16)
Lin et al. [124]	271,412 (208 cases)		11	home	1.24 (0.84–1.85)
Reynolds et al. [12]	57,523 (1754 cases)		10	Home, at work or social	1.13 (0.96–1.33)
Luo et al. [125]	41,022 (1660 cases)		10	home or at work	1.11 (0.92–1.34)
Xue et al. [126]	36,017 (2890 cases)		24	home or at work	0.97 (0.81–1.16)
Rosenberg et al. [127]	52,425 (1377 cases)		14	home	1.18 (0.98–1.42)
Dossus et al. [128]	183,608 (6264 cases)		11	home or at work	1.10 (1.01–1.20)
Wada et al. [129]	36, 990 (15,719 cases)		16	home	1.98 (1.03–3.84)
Case-control studies					
	Cases	Controls			
Sandler et al. [2]	518	518		home	1.8 (1.0–3.7)
Johnson et al. [130]	2317	2438		home or at work	Premenopausal women: 2.6 (1.1–6.0) Postmenopausal women: 1.1 (0.6–1.8)
Kropp et al. [131]	197	459		home, at work	1.6 (1.1–2.4)
Shrubsole et al. [132]	1013	1117		home, at work or outside of home	1.1 (0.8–1.4)
Bonner et al. [133]	1166	2105		home, at work	Premenopausal women: 1.17 (0.54–2.56) Postmenopausal women: 1.29 (0.82–2.01)
Lissowska et al. [134]	2386	2502		home or at work	1.11 (0.85–1.46)
Roddam et al. [135]	639	640		home	Premenopausal women: 0.89 (0.64–1.25)
Slattery et al. [136]	1527	1601		home or at work	Premenopausal women: 1.2 (0.6–2.7) Postmenopausal women: 1.0 (0.6–1.7)
Young et al. [137]	6235	6533		home or at work	0.97 (0.88–1.08)
Anderson et al. [56]	3101	3471		home, at work or in social	Premenopausal women: 1.61 (0.74–3.52) Postmenopausal women: 1.03 (0.69–1.55)
Gao et al. [65]	669	682		home	1.47 (1.18–1.84)
Hu et al. [138]	196	211		Home, at work	1.54 (0.94–2.52)
Tong et al. [139]	312	312		home	1.46 (1.05–2.03)
Pimhanam et al. [66]	444	444		Home, at work or public	2.27(1.30–3.98)
Nishino et al. [140]	773	2057		home or at work	1.13 (0.90–1.42)
Li et al. [141]	877	890		home, at work	2.17 1.45–3.23
Meta-analyses					
Johnson et al. [13]	19 cohort and case-control studies			home or at work	1.27 (1.11–1.45)
Lee et al. [142]	22 cohort and case-control studies			home or at work	1.54 (1.17–2.04)
Miller et al. [58]	19 cohort and case-control studies			home or at work	1.25 (1.08–1.44)
Pirie et al. [123]	8 cohort studies			Home or at work	1.01 (0.96–1.06)
	17 case-control studies			Home or at work	1.21(1.11–1.32)
Yang et al. [143]	10 cohort studies			home, at work or public	1.01 (0.96–1.06)
Chen et al. [144]	8 case-control studies			home	1.67(1.27–2.21)
Macacu et al. [145]	11 cohort and 20 case-control studies			Home or at work	1.20 (1.07–1.33)

## Nicotinic Acetylcholine Receptor (nAChRs) Signaling in Breast Cancer

### nAChRs

The nicotine contained in tobacco products and its receptor, nAChR, have been well studied due to their numerous effects on the human body [68–70]. This receptor family plays a role in the synthesis and release of neurotransmitters in the central nervous system and regulates all involuntary organ functions via the autonomic nervous system [71, 72]. The nAChR is a cation-selective channel receptor, and its activation is associated with cell proliferation, anti-apoptosis, and angiogenesis processes [73]. Moreover, many biological reports have suggested that the effects of tobacco exposure are mediated by the actions of nitrosamine and nAChRs [20, 74, 75].

Cancer related-nAChR function was first described in 1989 using small-cell lung cancer cell lines [76]. Although follow-up studies have supported that nicotine inhibited apoptosis via nAChRs in lung cancer cell lines, it has not been highlighted until the binding of nicotine-derived nitrosamine to α7nAChR was found because nicotine itself does not cause cancer in laboratory animals [20, 30, 77]. In 1998, Schuller finally discovered that the nicotine-derived nitrosamine and powerful carcinogen 4-(methylnitrosamino)-1-(3-pyridyl-1 butanone (NNK) is an α7nAChR agonist with a more than 1300-fold higher affinity for the α7nAChR than nicotine. This finding provided a completely new direction in tobacco-associated cancer research [20, 76].

### nAChRs in Cancers

The neuronal system expresses homo-pentamers (α7-α9 subunits) or hetero-pentamers (α2-α6, α10 subunits with β2-β4 subunits) of nAChRs, unlike the different combinations of nAChR subunits found in muscle (α1 with β1, γ, δ, or ε subunit) [78]. However, both arrangements are reportedly expressed and exhibit opposite functions in cancer cells. α7nAChR most effectively stimulates the growth of cancer cells, whereas α4β2nAChR inhibits growth [79, 80]. α7nAChR can bind to NNK with 1300 times higher affinity than to nicotine, whereas heteromeric αβ nAChRs exhibits a high affinity to NNN (~5000 times higher than to nicotine) [20, 21]. Notably, long-term exposure to nicotine and nicotine-derived nitrosamines, such as habitual tobacco smoking and exposure to SHS, upregulates all nAChRs but selectively leads the desensitization of α4β2nAChR activity, whereas the activity of α7nAChR remains unchanged [7]. Nicotine-derived nitrosamines change the expression and function of the α7nAChR to hyperactivate all its downstream “accelerator functions” while simultaneously activating desensitized heteromeric nAChRs, which disables their “brake” functions. Therefore, smoking is a prominent risk factor for all human cancers [80], and α7nAChR may represent a major receptor subfamily that mediates cancer cell proliferation.

Many studies have demonstrated that nAChR is closely involved in the development of various cancers [81]. The most well-studied nAChR mechanism is that of α7nAChR in tobacco-related non-small cell lung carcinoma (NSCLC). Specifically, Paleari et al. demonstrated that nicotinic receptor antagonists inhibit the growth of NSCLC, accounting for 30% of all primary pulmonary malignancies both in vitro and in vivo. [82]. Moreover, Improgo et al. showed that nAChR, especially α3β4α5 nAChR, plays a crucial role in small-cell lung cancer (SCLC). Using publicly available data of multiple genome-wide association studies (GWAS) in lung cancer, they found that gene variants encoding 3β4α5 nAChR are closely associated with lung cancer and confirmed that α3β4α5 nAChR promotes SCLC growth [22]. Nicotine also reportedly induces the secretion of different types of calpain, a proteolytic enzyme, from non-small cell lung cancer, which can promote the cleavage of various substrates in the extracellular matrix to result in metastasis and tumor progression [83]. In addition to lung cancer, the association of nicotine and nicotine derivatives with various types of nAChRs has been investigated in breast cancer [84, 85], colon cancer [86], leukemia [87, 88], cervical cancer [89], mesothelioma [90], medulloblastoma [91], and neuroblastoma [92].

### nAChR and Breast Cancer

Although α7nAChR is the oncogenic receptor responsible for most oncogenic responses in cancer, α9nAChR has recently become the focus of breast cancer research since α9nAChR is upregulated in estrogen receptor-positive breast cancer cells and stimulates the initiation and progression of breast cancer in coalition with the estrogen receptor [84]. Recent studies have documented that α9nAChR is highly associated with breast cancer [93–95]. Specifically, Chen et al. showed that the α9nAChR subunit played an important role in cell growth signaling in human breast cancer [95]. Lee et al. confirmed this finding both in vitro and in vivo to show that the down-regulation of α9nAChR results in decreased tumor volume for both the MDA-MB-231 human breast cancer cell line and xenografts in SCID mice [93]. Conversely, the stimulation of the α9nAChR led to breast cancer growth [96].

### nAChR Signaling in Cancers

The nAChR is the one of the best-characterized ligand-dependent cation channel receptors. nAChRs are expressed in autonomic ganglia and at the neuromuscular junction, and they play important roles in brain activity by being involved in synaptic and cellular function. Specifically, the opening of cation channel receptors results in the influx of sodium and calcium ions and exit of potassium ions, which causes membrane depolarization. [73].

Typically, nicotine- and NNK-mediated α7nAChR pathway activation has been observed to activate protein kinase A (PKA), the serine/threonine kinase Raf-1, the mitogen-activated kinases ERK1 and ERK2 and the transcription factors FOS, JUN and MYC in SCLC. [18, 22]. Moreover, PI3K-AKT and NF-kB activation by nicotine and NNK has been reported in NSCLC cell lines originating from large-cell carcinoma, squamous-cell carcinoma, and adenocarcinoma. Both pathways are known to be involved in cell proliferation [97]. In addition, Dasgupta et al. found that α3β4nAChR-initiated and XIAP- and survivin-mediated signaling play an important role in nicotine-induced chemoresistance in NSCLC. Moreover, PI3K-AKT and downstream XIAP or NF-kB signaling promote the anti-apoptotic effects of nicotine in A549 human adenocarcinoma cells, resulting in cell increased survival [98]. In other studies, both Mcl-1 and PPAR βδ proteins were also reported to be involved in nicotine-stimulated survival [99].

Notably, nAChR signaling affects other major characteristics of cancer development, such as angiogenesis, epithelial to mesenchymal transition (EMT) and metastasis. Furthermore, ERK/MAP kinase, PI3K-AKT and NF-kB signaling have been implicated in nicotine-induced and α7nAChR-mediated pro-angiogenic effects in endothelial cells. Specifically, Zhang et al. showed that nicotine induced pro-angiogenic effects by upregulating HIF-1α, and this effect was significantly attenuated by blocking the nAChR pathway, which consists of the Ca2+/calmodulin, Src, protein kinase C(PKC), PI3K-AKT, MAPK-ERK1/2 and mTOR pathways in NSCLC cell lines [100]. Additionally, the α7nAChR-mediated signaling pathway has recently been shown to be involved in the nicotine-induced invasion and migration of lung, breast and pancreatic cancer cells [98].

### nAChR Signaling in Breast Cancer

Although many studies have shown that nicotine and its derivatives from continuous tobacco smoking, including SHS, stimulate nAChRs to activate various intracellular signaling cascades that directly link to cancer characteristics (most were studied in lung cancer), little is known about the molecular mechanisms that connect nicotine to breast cancer.

In 2008, a signaling cascade involving PKC and cdc42 was identified in nicotine-induced mammary tumor migration. Guo et al. demonstrated that human breast cancer and normal cell lines constitutively express four types of nAChR subunits, and nicotine enhances the mobility of these cells. Thus, they concluded that nicotine initiates a signaling cascade that involves PKC and cdc42 and consequently promotes the migration of mammary epithelial or tumor cells by interacting with its receptor. However, they were not able to identify a receptor specific to breast cancer [83]

**Fig. 2:**
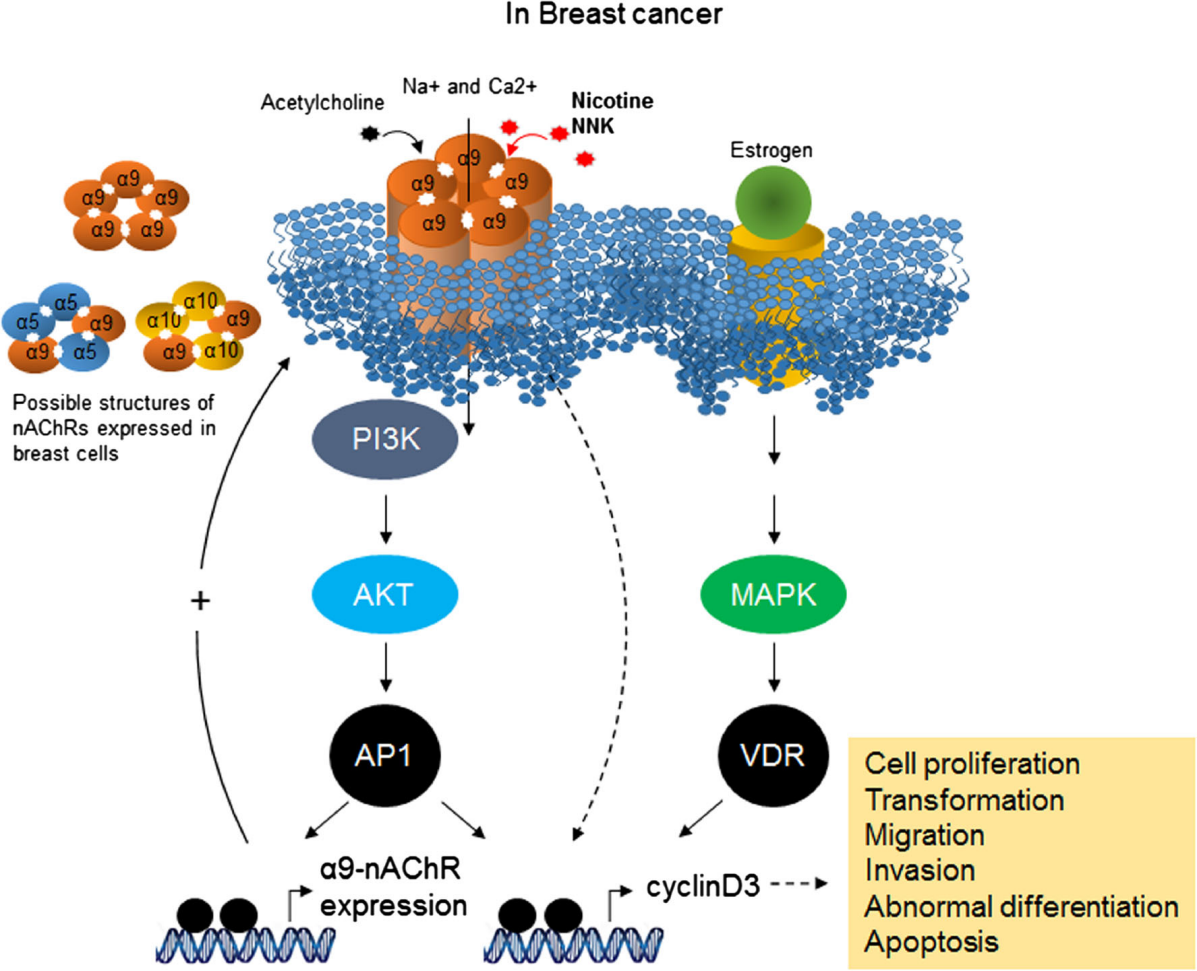
**α9nAChR involved signal pathway in breast cancer. **The three abundant α subunits expressed in breast tissue are able to form one homopentameric nAChR (α9nAChR) and two possible heteropentameric structures (α9α10-, α9α5-) of nAChRs. NNK and nicotine with estrogen stimulation can activate downstream transcription factors, AP1 and VDR through the PI3K/Akt and MAPK signaling pathways. The activation of AP1 and VDR transcription factors has been known by association with various cancer phenotypes including cell proliferation and migration. Binding of these transcription factors on the promoter region of α9-nAChR leads to a strong positive feedback loop

In 2010, Lee et al. identified α9nAChR as a nicotine-responsible receptor in breast cancer development [84, 93]. These studies found that α9nAChR plays a crucial role in human breast cancer cells that is mediated by nicotine and NNK. Moreover, they demonstrated that nicotine- and NNK-induced cancer cell proliferation was inhibited by knocking down α9nAChR using siRNA. By contrast, the over-expression of α9nAChR substantially increased tumor growth in a normal human breast cell line. Subsequently, a molecular pathway initiated by nicotine-mediated α9nAChR activation was studied in vivo and in vitro [101–103]. MAPK and PI3K-AKT can be activated by both nicotine and estrogen hormone stimulation. Importantly, the activation of α9nAChR features a strong positive feedback loop via the PI3K-AKT and MAPK pathways and is followed by the activation of AP1 and VDR transcription factors, which can bind to the promoter region of α9nAChR. These signal transduction cascades eventually result in cancer development [104] (Fig. 2). Additionally, acetylcholine and NNN have been demonstrated to exhibit a higher binding affinity to heteropentameric nAChRs (α4β2, α3β2, or α3β4) than to homopentameric nAChRs (α7, α9) [22, 105–107].

Indeed, the modulation of α9nAChR activity by nicotine and NNK in breast cells was associated with changes in proliferation, differentiation, migration, adhesion, cell contact, apoptosis, and angiogenesis [81].

## Conclusions

We herein showed that nicotine and its derivatives, i.e., NNK, in smoking tobacco products, and SHS are proven risk factors for cancer at all organ sites [108]. Mammary gland cancer is the most common cause of cancer death in females after lung cancer, and breast cancer caused by tobacco is referred to as tobacco-related primary and secondary cancer [109].

Long-term exposure to SHS among younger/primarily premenopausal women who had never smoked increased breast cancer risk by 60–70% because sidestream smoke contains more toxic chemicals than mainstream smoke [110].

The main component of tobacco smoke is nicotine, and it also contains more than 60 carcinogens. Nicotine is addictive but non-carcinogenic to healthy experimental animals, and the primary causes of cancer appear to be nicotine-derived nitrosamine and the powerful carcinogen NNK, which is an α7nAChR agonist with more than 1000-fold higher affinity to the receptor than nicotine. Additionally, it has been well documented that beta-adrenergic receptors and downstream cascades have a role in various cancer cell proliferation in response to NNK [111, 112]. Beta-adrenergic receptors, a family of G-protein coupled receptors, initiate multiple signaling cascades, including the adenylyl cyclase/cAMP/PKA/CREB pathway, which transactivates the epidermal growth factor receptor pathway [113–115].

Numerous studies have examined the role of nicotine and its receptors in the development of various cancers, especially lung cancer, but few articles have attempted to elucidate the molecular signaling cascades stimulated by NNK in breast cancer. Nevertheless, α9nAChR was identified as the receptor for nicotine and NNK in breast cells and is involved in nicotine-mediated cell proliferation in conjunction with estrogen hormone stimulation [93–95]

Although, all evidence provided in this review supports that SHS increases the risk of breast cancer development via NNK and its major target, the α9nAChR, extensive studies are necessary in the future to better understand the mechanism of tobacco-related breast cancer and assess the effect of low-dose exposures, such as that provided by SHS, on the metastatic process. Moreover, published data regarding the potential toxic effects of non-nicotine chemicals incorporated in tobacco smoke, which can also include hazardous substances, are limited. In addition, the measurement of chronic tobacco smoke exposure has become essential to study the metabolic fate of tobacco products and their potential impact on public health, particularly in non-smokers. For example, Pérez-Ortuño developed a method to analyze nicotine, cotinine, NNN, NNK, and NNAL in human hair samples. Although this study was subject to several limitations, such as hair composition (e.g., the color of hair, cosmetic treatments, etc.), they concluded that the detection of NNK instead of nicotine in hair is a very good biomarker of long-term exposure to SHS. Therefore, monitoring the hair NNK levels among non-smokers irregularly exposed to SHS may serve as a good biomarker of individual carcinogenic risk [116].

## Abbreviations

Carex: carcinogen exposure
EMT: epithelial to mesenchymal transition
ETS: environmental tobacco smoke
FDA: food and drug administration
GWAS: genome-wide association studies
IARC: international agency for research on cancer
nAChR: nicotine acetylcholine receptor
NDEA: N-Nitrosodiethylamine
NDELA: N-nitrosodiethanolamine
NDMA: N-nitrosodimethylamine
NMEA: N-nitrosomethylethylamine
NMOR: N-nitrosomorpholine
NNAL: 4-(Methylnitrosamino)-1-(3-pyridyl)-1-butanol
NNK: 4-(methylnitrosamino)-1-(3-pyridyl)-1-butanone
NNN: N-nitrosonornicotine
NPIP: N-nitrosopiperidine
NPYR: N-nitrosopyrrolidine
NSAR: N-nitrososarcosine
NSCLC: non-small cell lung carcinoma
PAHs: polycyclic aromatic hydrocarbons
PKA: protein kinase A
PKC: protein kinase C
SCLC: small-cell lung cancer
SHS: secondhand smoking

## References

[1] Hirayama T (2000). Non-smoking wives of heavy smokers have a higher risk of lung cancer: a study from Japan. 1981. Bull World Health Organ.

[2] SANDLER DP, EVERSON RB, WILCOX AJ. PASSIVE SMOKING IN ADULTHOOD AND CANCER RISK. Am J Epidemiol 1985;121(1):37–48. 10.1093/oxfordjournals.aje.a113980 3964991

[3] Stewart BW, Bray F, Forman D, Ohgaki H, Straif K, Ullrich A (2016). Cancer prevention as part of precision medicine: ‘plenty to be done’. Carcinogenesis.

[4] Stewart BW, Wild, C. P. World Cancer Report 2014. Lyon International Agency for Research on Cancer; 2014.

[5] Das S, Sen S, Mukherjee A, Chakraborty D, Mondal PK (2012). Risk factors of breast cancer among women in eastern India: a tertiary hospital based case control study. Asian Pac J Cancer Prev.

[6] Majeed WAB, Javed I, Khaliq T, Muhammad F, Ali A, Raza A (2014). Breast cancer: major risk factors and recent developments in treatment. Asian Pac J Cancer Prev.

[7] FactorsinBreast_Cancer CGoH. Alcohol, tobacco and breast cancer - collaborative reanalysis of individual data from 53 epidemiological studies, including 58 515 women with breast cancer and 95 067 women without the disease. Br J Cancer 2002;87(11):1234–1245. http://www.nature.com/bjc/journal/v87/n11/suppinfo/6600596s1.html. 10.1038/sj.bjc.6600596 PMC2562507 12439712

[8] Chu SY, Stroup NE, Wingo PA, Lee NC, Peterson HB, Gwinn ML (1990). Cigarette smoking and the risk of breast cancer. Am J Epidemiol.

[9] Cal E. Proposed identification of environmental tobacco smoke as a toxic air contaminant. Part a: Exposure assessment. 2005;

[10] Agency CEP (1997). Health effects of exposure to environmental tobacco smoke. California Environmental Protection Agency Tob Control.

[11] Kauppinen T, Toikkanen J, Pedersen D, Young R, Ahrens W, Boffetta P (2000). Occupational exposure to carcinogens in the European Union. Occup Environ Med.

[12] Reynolds P, Goldberg D, Hurley S, Nelson DO, Largent J, Henderson KD (2009). Passive smoking and risk of breast cancer in the California teachers study. Cancer epidemiology, biomarkers & prevention : a publication of the American Association for Cancer Research, cosponsored by the American Society of Preventive Oncology.

[13] Johnson KC (2005). Accumulating evidence on passive and active smoking and breast cancer risk. Int J Cancer.

[14] Horton AW (1988). Indoor tobacco smoke pollution. A major risk factor for both breast and lung cancer?. Cancer.

[15] Johnson KC, Glantz SA (2008). Evidence secondhand smoke causes breast cancer in 2005 stronger than for lung cancer in 1986. Prev Med.

[16] Hoffmann D, Hoffmann I, El-Bayoumy K (2001). The less harmful cigarette: a controversial issue. A tribute to Ernst L. Wynder. Chem Res Toxicol.

[17] Rudel RA, Attfield KR, Schifano JN, Brody JG (2007). Chemicals causing mammary gland tumors in animals signal new directions for epidemiology, chemicals testing, and risk assessment for breast cancer prevention. Cancer.

[18] Wynder EL, Muscat JE (1995). The changing epidemiology of smoking and lung cancer histology. Environ Health Perspect.

[19] Proctor RN (2002). Tobacco and the global epidemic of lung cancer. Cas Lek Cesk.

[20] Schuller HM, Orloff M (1998). Tobacco-specific carcinogenic nitrosamines: ligands for nicotinic acetylcholine receptors in human lung cancer cells. Biochem Pharmacol.

[21] Schuller HM (2007). Nitrosamines as nicotinic receptor ligands. Life Sci.

[22] Improgo MR, Soll LG, Tapper AR, Gardner PD (2013). Nicotinic acetylcholine receptors mediate lung cancer growth. Front Physiol.

[23] Jenkins RA, Tomkins B, Guerin MR. The chemistry of environmental tobacco smoke: composition and measurement: CRC Press; 2000.

[24] Health UDo. Services H. The health consequences of involuntary exposure to tobacco smoke: a report of the Surgeon General. Atlanta, GA: US Department of Health and Human Services, Centers for Disease Control and Prevention, coordinating Center for Health Promotion, National Center for Chronic Disease Prevention and Health Promotion, Office on smoking and. Health. 2006;709 20669524

[25] Rodgman A (2013). Perfetti TA.

[26] Byrd III DM. REVIEW OF INGREDIENTS ADDED TO CIGARETTES PHASE ONE: The feasibility of testing ingredients added to cigarettes. 2004.

[27] Oldham MJ, DeSoi DJ, Rimmer LT, Wagner KA, Morton MJ (2014). Insights from analysis for harmful and potentially harmful constituents (HPHCs) in tobacco products. Regul Toxicol Pharmacol.

[28] Zhou G, Xiao W, Xu C, Hu Y, Wu X, Huang F (2016). Chemical constituents of tobacco smoke induce the production of interleukin-8 in human bronchial epithelium, 16HBE cells. Tob Induc Dis.

[29] Witschi H, Espiritu I, Maronpot RR, Pinkerton KE, Jones AD (1997). The carcinogenic potential of the gas phase of environmental tobacco smoke. Carcinogenesis.

[30] Hecht SS, Chen CB, Hirota N, Ornaf RM, Tso TC, Hoffmann D (1978). Tobacco-specific nitrosamines: formation from nicotine in vitro and during tobacco curing and carcinogenicity in strain a mice. J Natl Cancer Inst.

[31] Coffa BG, Coggins CR, Werley MS, Oldham MJ, Fariss MW (2016). Chemical, physical, and in vitro characterization of research cigarettes containing denicotinized tobacco. Regulatory toxicology and pharmacology : RTP.

[32] Peterson LA (2017). Context matters: contribution of specific DNA adducts to the genotoxic properties of the tobacco-specific nitrosamine NNK. Chem Res Toxicol.

[33] Hukkanen J, Jacob P, 3rd, Benowitz NL. Metabolism and disposition kinetics of nicotine. Pharmacol Rev 2005;57(1):79–115. doi:10.1124/pr.57.1.3. 10.1124/pr.57.1.3 15734728

[34] Humans IWGotEoCRt. Tobacco smoke and involuntary smoking. IARC Monogr Eval Carcinog Risks Hum 2004;83:1–1438. PMC4781536 15285078

[35] Hoffmann D, Hecht SS (1985). Nicotine-derived N-nitrosamines and tobacco-related cancer: current status and future directions. Cancer Res.

[36] IARC. Smokeless Tobacco and Some Tobacco-specific N-Nitrosamines. IARC Monographs on the Evaluation of Carcinogenic Risks to Humans; IARC. Lyon, France: 2007. PMC4781254 18335640

[37] Hoffmann D, Brunnemann KD, Adams JD, Hecht SS (1984). Formation and analysis of N-nitrosamines in tobacco products and their endogenous formation in consumers. IARC Sci Publ.

[38] Adams JD, Lee SJ, Vinchkoski N, Castonguay A, Hoffmann D (1983). On the formation of the tobacco-specific carcinogen 4-(methylnitrosamino)-1-(3-pyridyl)-1-butanone during smoking. Cancer Lett.

[39] Upadhyaya P, Kenney PM, Hochalter JB, Wang M, Hecht SS (1999). Tumorigenicity and metabolism of 4-(methylnitrosamino)-1-(3-pyridyl)-1-butanol enantiomers and metabolites in the a/J mouse. Carcinogenesis.

[40] Doll R, Peto R (1981). The causes of cancer: quantitative estimates of avoidable risks of cancer in the United States today. J Natl Cancer Inst.

[41] Centers for Disease C. The Surgeon General's 1989 Report on Reducing the Health Consequences of Smoking: 25 Years of Progress. MMWR Suppl. 1989;38(2):1–32. 2494426

[42] Secretan B, Straif K, Baan R, Grosse Y, El Ghissassi F, Bouvard V (2009). A review of human carcinogens--part E: tobacco, areca nut, alcohol, coal smoke, and salted fish. Lancet Oncol.

[43] Centers for Disease C, Prevention, National Center for Chronic Disease P, Health P, Office on S, Health. Publications and Reports of the Surgeon General. How Tobacco Smoke Causes Disease: The Biology and Behavioral Basis for Smoking-Attributable Disease: A Report of the Surgeon General. Atlanta (GA): Centers for Disease Control and Prevention (US); 2010. 21452462

[44] Autrup H, Stoner GD (1982). Metabolism of N-nitrosamines by cultured human and rat esophagus. Cancer Res.

[45] Carmella SG, Han S, Villalta PW, Hecht SS (2005). Analysis of total 4-(methylnitrosamino)-1-(3-pyridyl)-1-butanol in smokers' blood. Cancer epidemiology, biomarkers & prevention : a publication of the American Association for Cancer Research, cosponsored by the American Society of Preventive Oncology..

[46] Carmella SG, Borukhova A, Akerkar SA, Hecht SS (1997). Analysis of human urine for pyridine-N-oxide metabolites of 4-(methylnitrosamino)-1-(3-pyridyl)-1-butanone, a tobacco-specific lung carcinogen. Cancer epidemiology, biomarkers & prevention : a publication of the American Association for Cancer Research, cosponsored by the American Society of Preventive Oncology..

[47] Carmella SG, Kagan SS, Kagan M, Foiles PG, Palladino G, Quart AM (1990). Mass spectrometric analysis of tobacco-specific nitrosamine hemoglobin adducts in snuff dippers, smokers, and nonsmokers. Cancer Res.

[48] Klus H (1990). Distribution of mainstream and Sidestream cigarette smoke components. Recent Advances in Tobacco Science.

[49] Goniewicz ML, Knysak J, Gawron M, Kosmider L, Sobczak A, Kurek J (2014). Levels of selected carcinogens and toxicants in vapour from electronic cigarettes. Tob Control.

[50] Farsalinos KE, Gillman G, Poulas K, Voudris V (2015). Tobacco-specific nitrosamines in electronic cigarettes: comparison between liquid and aerosol levels. Int J Environ Res Public Health.

[51] Miller GH (1990). The impact of passive smoking: cancer deaths among nonsmoking women. Cancer Detect Prev.

[52] Boone SD, Baumgartner KB, Baumgartner RN, Connor AE, John EM, Giuliano AR (2015). Active and passive cigarette smoking and mortality among Hispanic and non-Hispanic white women diagnosed with invasive breast cancer. Ann Epidemiol.

[53] Band PR, Le ND, Fang R, Deschamps M (2002). Carcinogenic and endocrine disrupting effects of cigarette smoke and risk of breast cancer. Lancet.

[54] Hanaoka T, Yamamoto S, Sobue T, Sasaki S, Tsugane S (2005). Japan public health Center-based prospective study on C et al. active and passive smoking and breast cancer risk in middle-aged Japanese women. Int J Cancer.

[55] Johnson KC, Miller AB, Collishaw NE, Palmer JR, Hammond SK, Salmon AG (2011). Active smoking and secondhand smoke increase breast cancer risk: the report of the Canadian expert panel on tobacco smoke and breast cancer risk (2009). Tob Control.

[56] Anderson LN, Cotterchio M, Mirea L, Ozcelik H, Kreiger N (2012). Passive cigarette smoke exposure during various periods of life, genetic variants, and breast cancer risk among never smokers. Am J Epidemiol.

[57] Li D, Zhang W, Sahin AA, Hittelman WN (1999). DNA adducts in normal tissue adjacent to breast cancer: a review. Cancer Detect Prev.

[58] Miller MD, Marty MA, Broadwin R, Johnson KC, Salmon AG, Winder B (2007). The association between exposure to environmental tobacco smoke and breast cancer: a review by the California Environmental Protection Agency. Prev Med.

[59] Reynolds P (2013). Smoking and breast cancer. J Mammary Gland Biol Neoplasia.

[60] Wells AJ (1991). Breast cancer, cigarette smoking, and passive smoking. Am J Epidemiol.

[61] Schick S, Glantz S (2005). Philip Morris toxicological experiments with fresh sidestream smoke: more toxic than mainstream smoke. Tob Control.

[62] Fischer S, Castonguay A, Kaiserman M, Spiegelhalder B, Preussmann R (1990). Tobacco-specific nitrosamines in Canadian cigarettes. J Cancer Res Clin Oncol.

[63] Luo J, Margolis KL, Wactawski-Wende J, Horn K, Messina C, Stefanick ML (2011). Association of active and passive smoking with risk of breast cancer among postmenopausal women: a prospective cohort study. BMJ.

[64] Jee SH, Ohrr H, Kim IS (1999). Effects of husbands' smoking on the incidence of lung cancer in Korean women. Int J Epidemiol.

[65] Gao CM, Ding JH, Li SP, Liu YT, Qian Y, Chang J (2013). Active and passive smoking, and alcohol drinking and breast cancer risk in chinese women. Asian Pac J Cancer Prev.

[66] Pimhanam C, Sangrajrang S, Ekpanyaskul C (2014). Tobacco smoke exposure and breast cancer risk in Thai urban females. Asian Pac J Cancer Prev.

[67] Piazza KM, Hyland A (2011). Prevalence of rules prohibiting home and workplace smoking correlates with state-specific breast cancer outcomes: an ecologic analysis. Tob Control.

[68] Dang N, Meng X, Song H (2016). Nicotinic acetylcholine receptors and cancer (review). Biomedical reports.

[69] Albuquerque EX, Pereira EF, Alkondon M, Rogers SW (2009). Mammalian nicotinic acetylcholine receptors: from structure to function. Physiol Rev.

[70] Sargent PB (1993). The diversity of neuronal nicotinic acetylcholine receptors. Annu Rev Neurosci.

[71] Gotti C, Fornasari D, Clementi F (1997). Human neuronal nicotinic receptors. Prog Neurobiol.

[72] Grando SA, Kawashima K, Wessler I (2003). Introduction: the non-neuronal cholinergic system in humans. Life Sci.

[73] Zdanowski R, Krzyzowska M, Ujazdowska D, Lewicka A, Lewicki S (2015). Role of alpha7 nicotinic receptor in the immune system and intracellular signaling pathways. Cent Eur J Immunol.

[74] Al-Wadei HA, Majidi M, Tsao MS, Schuller HM (2007). Low concentrations of beta-carotene stimulate the proliferation of human pancreatic duct epithelial cells in a PKA-dependent manner. Cancer Genomics Proteomics.

[75] Jull B, Plummer H, Schuller H (2001). Nicotinic receptor-mediated activation by the tobacco-specific nitrosamine NNK of a Raf-1/MAP kinase pathway, resulting in phosphorylation of c-myc in human small cell lung carcinoma cells and pulmonary neuroendocrine cells. J Cancer Res Clin Oncol.

[76] Schuller HM (1989). Cell type specific, receptor-mediated modulation of growth kinetics in human lung cancer cell lines by nicotine and tobacco-related nitrosamines. Biochem Pharmacol.

[77] Hecht SS, Hoffmann D (1988). Tobacco-specific nitrosamines, an important group of carcinogens in tobacco and tobacco smoke. Carcinogenesis.

[78] Walke W, Staple J, Adams L, Gnegy M, Chahine K, Goldman D (1994). Calcium-dependent regulation of rat and chick muscle nicotinic acetylcholine receptor (nAChR) gene expression. J Biol Chem.

[79] Wang J, Wang Y, Wang Y, Wang R, Zhang Y, Zhang Q (2014). Contribution of alpha4beta2 nAChR in nicotine-induced intracellular calcium response and excitability of MSDB neurons. Brain Res.

[80] Schuller HM (2012). Regulatory role of the alpha7nAChR in cancer. Curr Drug Targets.

[81] Grando SA (2014). Connections of nicotine to cancer. Nat Rev Cancer.

[82] Paleari L, Catassi A, Ciarlo M, Cavalieri Z, Bruzzo C, Servent D (2008). Role of α7-nicotinic acetylcholine receptor in human non-small cell lung cancer proliferation. Cell Prolif.

[83] Guo J, Ibaragi S, Zhu T, Luo L-Y, Hu G-F, Huppi PS (2008). Nicotine promotes mammary tumor migration via a signaling cascade involving protein kinase C and CDC42. Cancer Res.

[84] Lee C-H, Chang Y-C, Chen C-S, Tu S-H, Wang Y-J, Chen L-C (2011). Crosstalk between nicotine and estrogen-induced estrogen receptor activation induces α9-nicotinic acetylcholine receptor expression in human breast cancer cells. Breast Cancer Res Treat.

[85] Linnoila RI (2010). From nicotine to breast cancer, implications of cholinergic receptor pathway. J Natl Cancer Inst.

[86] Ye YN, Liu ES, Shin VY, Wu WK, Cho CH (2004). The modulating role of nuclear factor-kappaB in the action of alpha7-nicotinic acetylcholine receptor and cross-talk between 5-lipoxygenase and cyclooxygenase-2 in colon cancer growth induced by 4-(N-methyl-N-nitrosamino)-1-(3-pyridyl)-1-butanone. J Pharmacol Exp Ther.

[87] Sato KZ, Fujii T, Watanabe Y, Yamada S, Ando T, Kazuko F (1999). Diversity of mRNA expression for muscarinic acetylcholine receptor subtypes and neuronal nicotinic acetylcholine receptor subunits in human mononuclear leukocytes and leukemic cell lines. Neurosci Lett.

[88] Codignola A, Tarroni P, Cattaneo MG, Vicentini LM, Clementi F, Sher E (1994). Serotonin release and cell proliferation are under the control of alpha-bungarotoxin-sensitive nicotinic receptors in small-cell lung carcinoma cell lines. FEBS Lett.

[89] Calleja-Macias IE, Kalantari M, Bernard HU (2009). Cholinergic signaling through nicotinic acetylcholine receptors stimulates the proliferation of cervical cancer cells: an explanation for the molecular role of tobacco smoking in cervical carcinogenesis?. Int J Cancer.

[90] Trombino S, Cesario A, Margaritora S, Granone P, Motta G, Falugi C (2004). Alpha7-nicotinic acetylcholine receptors affect growth regulation of human mesothelioma cells: role of mitogen-activated protein kinase pathway. Cancer Res.

[91] Siegel HN, Lukas RJ (1988). Nicotinic agonists regulate alpha-bungarotoxin binding sites of TE671 human medulloblastoma cells. J Neurochem.

[92] Lukas RJ (1993). Expression of ganglia-type nicotinic acetylcholine receptors and nicotinic ligand binding sites by cells of the IMR-32 human neuroblastoma clonal line. J Pharmacol Exp Ther.

[93] Lee CH, Huang CS, Chen CS, Tu SH, Wang YJ, Chang YJ (2010). Overexpression and activation of the alpha9-nicotinic receptor during tumorigenesis in human breast epithelial cells. J Natl Cancer Inst.

[94] Chen CS, Lee CH, Hsieh CD, Ho CT, Pan MH, Huang CS (2011). Nicotine-induced human breast cancer cell proliferation attenuated by garcinol through down-regulation of the nicotinic receptor and cyclin D3 proteins. Breast Cancer Res Treat.

[95] Shih YL, Liu HC, Chen CS, Hsu CH, Pan MH, Chang HW (2010). Combination treatment with luteolin and quercetin enhances antiproliferative effects in nicotine-treated MDA-MB-231 cells by down-regulating nicotinic acetylcholine receptors. J Agric Food Chem.

[96] Zhao Y (2016). The oncogenic functions of nicotinic acetylcholine receptors. Journal of Oncology.

[97] Wen J, Fu JH, Zhang W, Guo M (2011). Lung carcinoma signaling pathways activated by smoking. Chinese journal of cancer.

[98] Dasgupta P, Rizwani W, Pillai S, Kinkade R, Kovacs M, Rastogi S (2009). Nicotine induces cell proliferation, invasion and epithelial-mesenchymal transition in a variety of human cancer cell lines. Int J Cancer.

[99] Cardinale A, Nastrucci C, Cesario A, Russo P (2012). Nicotine: specific role in angiogenesis, proliferation and apoptosis. Crit Rev Toxicol.

[100] Singh S, Pillai S, Chellappan S. Nicotinic acetylcholine receptor signaling in tumor growth and metastasis. Journal of oncology. 2011;2011 10.1155/2011/456743 PMC3085312 21541211

[101] Callaghan B, Adams DJ (2010). Analgesic α-conotoxins Vc1. 1 and Rg1A inhibit N-type calcium channels in sensory neurons of α9 nicotinic receptor knockout mice. Channels.

[102] Ho Y-S, Lee C-H, Wu C-H (2011). The alpha 9-nicotinic acetylcholine receptor serves as a molecular target for breast cancer therapy. Journal of Experimental & Clinical Medicine.

[103] Katz E, Verbitsky M, Rothlin CV, Vetter DE, Heinemann SF, Elgoyhen AB (2000). High calcium permeability and calcium block of the α9 nicotinic acetylcholine receptor. Hear Res.

[104] Guha P, Bandyopadhyaya G, Polumuri SK, Chumsri S, Gade P, Kalvakolanu DV (2014). Nicotine promotes apoptosis resistance of breast cancer cells and enrichment of side population cells with cancer stem cell-like properties via a signaling cascade involving galectin-3, α9 nicotinic acetylcholine receptor and STAT3. Breast Cancer Res Treat.

[105] Wang F, Gerzanich V, Wells GB, Anand R, Peng X, Keyser K (1996). Assembly of human neuronal nicotinic receptor alpha5 subunits with alpha3, beta2, and beta4 subunits. J Biol Chem.

[106] Groot-Kormelink PJ, Boorman JP, Sivilotti LG (2001). Formation of functional alpha3beta4alpha5 human neuronal nicotinic receptors in Xenopus oocytes: a reporter mutation approach. Br J Pharmacol.

[107] Ramirez-Latorre J, Yu CR, Qu X, Perin F, Karlin A, Role L (1996). Functional contributions of alpha5 subunit to neuronal acetylcholine receptor channels. Nature.

[108] Health UDo (2014). Services H. Let's make the next generation tobacco-free: your guide to the 50th anniversary Surgeon General's report on smoking and health.

[109] Catsburg C, Kirsh VA, Soskolne CL, Kreiger N, Rohan TE (2014). Active cigarette smoking and the risk of breast cancer: a cohort study. Cancer Epidemiol.

[110] Schick S, Glantz SA (2006). Sidestream cigarette smoke toxicity increases with aging and exposure duration. Tob Control.

[111] Weddle DL, Tithoff P, Williams M, Schuller HM (2001). Beta-adrenergic growth regulation of human cancer cell lines derived from pancreatic ductal carcinomas. Carcinogenesis.

[112] Schuller HM, Cole B (1989). Regulation of cell proliferation by beta-adrenergic receptors in a human lung adenocarcinoma cell line. Carcinogenesis.

[113] Slomiany BL, Slomiany A (2004). Src-kinase-dependent epidermal growth factor receptor transactivation in salivary mucin secretion in response to beta-adrenergic G-protein-coupled receptor activation. Inflammopharmacology.

[114] Dorsam RT, Gutkind JS (2007). G-protein-coupled receptors and cancer. Nat Rev Cancer.

[115] Schuller HM, Tithof PK, Williams M, Plummer H, 3rd. The tobacco-specific carcinogen 4-(methylnitrosamino)-1-(3-pyridyl)-1-butanone is a beta-adrenergic agonist and stimulates DNA synthesis in lung adenocarcinoma via beta-adrenergic receptor-mediated release of arachidonic acid. Cancer Res 1999;59(18):4510–4515. 10493497

[116] Perez-Ortuno R, Martinez-Sanchez JM, Fu M, Fernandez E, Pascual JA (2016). Evaluation of tobacco specific nitrosamines exposure by quantification of 4-(methylnitrosamino)-1-(3-pyridyl)-1-butanone (NNK) in human hair of non-smokers. Scientific reports.

[117] Cressey D (2013). Regulation stacks up for e-cigarettes. Nature.

[118] Grana R, Benowitz N, Glantz SA (2014). E-cigarettes: a scientific review. Circulation.

[119] Fischer S, Spiegelhalder B, Preussmann R (1990). Tobacco-specific nitrosamines in European and USA cigarettes. Archiv fur Geschwulstforschung.

[120] Hoffmann D, Adams JD, Brunnemann KD, Hecht SS (1979). Assessment of tobacco-specific N-nitrosamines in tobacco products. Cancer Res.

[121] Egan KM, Stampfer MJ, Hunter D, Hankinson S, Rosner BA, Holmes M (2002). Active and passive smoking in breast cancer: prospective results from the Nurses' health study. Epidemiology.

[122] Reynolds P, Hurley S, Goldberg DE, Anton-Culver H, Bernstein L, Deapen D (2004). Active smoking, household passive smoking, and breast cancer: evidence from the California teachers study. J Natl Cancer Inst.

[123] Pirie K, Beral V, Peto R, Roddam A, Reeves G, Green J (2008). Passive smoking and breast cancer in never smokers: prospective study and meta-analysis. Int J Epidemiol.

[124] Lin Y, Kikuchi S, Tamakoshi K, Wakai K, Kondo T, Niwa Y (2008). Active smoking, passive smoking, and breast cancer risk: findings from the Japan collaborative cohort study for Evaluation of cancer risk. J Epidemiol.

[125] Luo J, Horn K, Ockene JK, Simon MS, Stefanick ML, Tong E (2011). Interaction between smoking and obesity and the risk of developing breast cancer among postmenopausal women: the Women's Health Initiative observational study. Am J Epidemiol.

[126] Xue F, Willett WC, Rosner BA, Hankinson SE, Michels KB (2011). Cigarette smoking and the incidence of breast cancer. Arch Intern Med.

[127] Rosenberg L, Boggs DA, Bethea TN, Wise LA, Adams-Campbell LL, Palmer JR (2013). A prospective study of smoking and breast cancer risk among African-American women. Cancer Causes Control.

[128] Dossus L, Boutron-Ruault MC, Kaaks R, Gram IT, Vilier A, Fervers B (2014). Active and passive cigarette smoking and breast cancer risk: results from the EPIC cohort. Int J Cancer.

[129] Wada K, Kawachi T, Hori A, Takeyama N, Tanabashi S, Matsushita S (2015). Husband's smoking status and breast cancer risk in Japan: from the Takayama study. Cancer Sci.

[130] Johnson KC, Hu J, Mao Y (2000). Canadian cancer registries epidemiology research G. Passive and active smoking and breast cancer risk in Canada, 1994-97. Cancer Causes Control.

[131] Kropp S, Chang-Claude J (2002). Active and passive smoking and risk of breast cancer by age 50 years among German women. Am J Epidemiol.

[132] Shrubsole MJ, Gao YT, Dai Q, Shu XO, Ruan ZX, Jin F (2004). Passive smoking and breast cancer risk among non-smoking Chinese women. Int J Cancer.

[133] Bonner MR, Nie J, Han D, Vena JE, Rogerson P, Muti P (2005). Secondhand smoke exposure in early life and the risk of breast cancer among never smokers (United States). Cancer Causes Control.

[134] Lissowska J, Brinton LA, Zatonski W, Blair A, Bardin-Mikolajczak A, Peplonska B (2006). Tobacco smoking, NAT2 acetylation genotype and breast cancer risk. Int J Cancer.

[135] Roddam AW, Pirie K, Pike MC, Chilvers C, Crossley B, Hermon C (2007). Active and passive smoking and the risk of breast cancer in women aged 36-45 years: a population based case-control study in the UK. Br J Cancer.

[136] Slattery ML, Curtin K, Giuliano AR, Sweeney C, Baumgartner R, Edwards S (2008). Active and passive smoking, IL6, ESR1, and breast cancer risk. Breast Cancer Res Treat.

[137] Young E, Leatherdale S, Sloan M, Kreiger N, Barisic A (2009). Age of smoking initiation and risk of breast cancer in a sample of Ontario women. Tob Induc Dis.

[138] Hu M, Han D, Sun S, Yan Y, Zhang J, Zhou Y (2013). Bleomycin-induced mutagen sensitivity, passive smoking, and risk of breast cancer in Chinese women: a case-control study. Cancer Causes Control.

[139] Tong J-h, Li Z, Shi J, Li H-m, Wang Y, Fu L-y et al. Passive Smoking Exposure from Partners as a Risk Factor for ER+/PR+ Double Positive Breast Cancer in Never-Smoking Chinese Urban Women: A Hospital-Based Matched Case Control Study. PLoS ONE. 2014;9(5):e97498. doi:10.1371/journal.pone.0097498. 10.1371/journal.pone.0097498 PMC4035255 24866166

[140] Nishino Y, Minami Y, Kawai M, Fukamachi K, Sato I, Ohuchi N (2014). Cigarette smoking and breast cancer risk in relation to joint estrogen and progesterone receptor status: a case-control study in Japan. Spring.

[141] Li B, Wang L, Lu MS, Mo XF, Lin FY, Ho SC (2015). Passive smoking and breast cancer risk among non-smoking women: a case-control study in China. PLoS One.

[142] Lee PN, Hamling J (2006). Environmental tobacco smoke exposure and risk of breast cancer in nonsmoking women: a review with meta-analyses. Inhal Toxicol.

[143] Yang Y, Zhang F, Skrip L, Wang Y, Liu S (2013). Lack of an association between passive smoking and incidence of female breast cancer in non-smokers: evidence from 10 prospective cohort studies. PLoS One.

[144] Chen Z, Shao J, Gao X, Li X. Effect of passive smoking on female breast cancer in China: a meta-analysis. Asia Pac J Public Health. 2015;27(2):Np58–64. doi:10.1177/1010539513481493. 10.1177/1010539513481493 23572370

[145] Macacu A, Autier P, Boniol M, Boyle P (2015). Active and passive smoking and risk of breast cancer: a meta-analysis. Breast Cancer Res Treat.

